# Health care seeking among pulmonary tuberculosis suspects and patients in rural Ethiopia: a community-based study

**DOI:** 10.1186/1471-2458-9-454

**Published:** 2009-12-09

**Authors:** Solomon Yimer, Carol Holm-Hansen, Tilahun Yimaldu, Gunnar Bjune

**Affiliations:** 1 Department of General Practice and Community Medicine, University of Oslo, Oslo, Norway; 2 Amhara Regional State Health Bureau, Bahir Dar, Ethiopia; 3 Norwegian Institute of Public Health, Oslo, Norway

## Abstract

**Background:**

Health care seeking is a dynamic process that is influenced by socio-demographic, cultural and other factors. In Ethiopia, there are limited studies regarding the health seeking behaviour of tuberculosis (TB) suspects and TB patients. However, a thorough understanding of patients' motivation and actions is crucial to understanding TB and the treatment of disease. Such insights would conceivably help to reduce delay in diagnosis, improve treatment adherence and thereby reduce transmission of TB in the community. The objective of this study was to describe and analyze health care seeking among TB suspects and pulmonary TB (PTB) cases in a rural district of the Amhara Region in Ethiopia.

**Methods:**

Study *kebeles *were randomly selected in a cross-sectional study design. House-to-house visits were conducted in which individuals aged 15 years and above in all households of the *kebeles *were included. Subjects with symptoms suggestive of TB were interviewed about their health seeking behaviour, socio-demographic and clinical factors using a semi-structured questionnaire. Logistics regression analysis was employed to assess associations between the independent and outcome variables.

**Results:**

The majority, 787 (78%), TB suspects and 33 (82.5%) PTB cases had taken health care actions for symptoms from sources outside their homes. The median delay before the first action was 30 days. In logistics regression, women (AOR 0.8, 95% CI 0.6, 0.9) were found to be less likely to visit a medical health provider than men. Those with a long duration of cough (AOR 1.5, 95% CI 1.03, 2.1) and those with a previous history of TB (AOR 1.5, 95% CI 1.03, 2.3) were more likely to visit a medical health provider compared to those with a shorter duration of cough and with no history of TB.

**Conclusion:**

The majority of TB suspects and PTB cases had already taken health care actions for their symptoms at the time of the survey. The availability of a simple and rapid diagnostic TB test for use at the lowest level of health care and the involvement of all health providers in case finding activities are imperative for early TB case detection.

## Background

Ethiopia ranks 7^th ^among the 22 countries with the highest tuberculosis (TB) burden in the world. The TB case detection rate is very low compared to the World Health Organization (WHO) target of detecting 70% of infectious TB cases [1]. Factors contributing to the low case detection rate of TB may be related to diagnostic delay resulting from the patients' failure to seek health care or the inability of the health system to detect TB at the community level [2].

Diagnostic delay in TB results in more severe illness and increased transmission of TB in the community. Understanding the diagnostic process and health seeking behaviour of TB patients is therefore necessary to improve case detection and provide timely treatment of TB. In this regard, a number of health facility-based studies have explored the duration of delay and factors related to health seeking among PTB patients [2]. However, too few community-based studies have been conducted to provide an understanding of health seeking behaviour among TB suspects [3-5]. As these groups of patients constitute pools from which infectious TB cases are detected, analysing their health seeking behaviour is very important to elucidate the magnitude and reasons for diagnostic delay in TB.

Various factors may affect the health care seeking behaviour of TB suspects. According to the literature, health care seeking is a dynamic process that is influenced by socio-demographic, cultural and other factors [6]. These factors affect the way individual patients perceive their symptoms, create concepts and make decisions regarding their health care actions. Therefore, a thorough analysis and understanding of the factors related to health care seeking from these perspectives is warranted in order to improve TB control programs.

In Ethiopia, no previous study has investigated the health seeking behaviour of TB suspects in particular at the community level. While many studies [7-9] have investigated the health seeking behaviour of TB patients in Ethiopia, such studies focused on TB patients that were already detected through the formal health system following passive case detection strategy. The health seeking behaviour of TB suspects and patients who prefer to stay at home despite having TB symptoms, or visit alternative sources of health care may be different compared to those who visit the formal medical providers. Such evidence can only be generated from community-based studies. This study therefore aims at describing and analyzing the factors of health care seeking behaviour among TB suspects and patients in a rural district of the Amhara Region in Ethiopia.

## Methods

### Study setting

The study was conducted during March 2008 in the Merawi district, West Gojam Zone of the Amhara Region in Ethiopia. The district lies 40 km from the regional capital, Bahir Dar, and the total population is estimated to be 292,250 with a male/female ratio of 1:1 [10]. Having a stable rural population, the district has three health centres, 16 health posts and is comprised of 43 *kebeles *(smallest administrative units). Only one of the health centres has facilities for performing sputum smear microscopy. The health service coverage is 62% and the DOTS strategy has been implemented to treat and control TB [11].

We followed a cluster sampling procedure to select the study *kebeles *(clusters). Twelve (29%) of the 43 *kebeles *of the district were randomly selected and included in the study. The 12 *kebeles *had a population of 81,468 with 47,478 (58.2%) above 15 years of age (Table 1). The sample size was calculated using a formula to estimate the prevalence of TB in a population [12]. By taking a previous study that showed a TB prevalence in Ethiopia of 189/100,000 population [13], a 95% confidence interval, margin error of 0.1% and design effect of 2, the minimum sample size was calculated to be 14,598 individuals. This is equivalent to 3,650 households with an estimated 4 adults/household.

**Table 1:** Socio-demographic characteristics of the study population in Merawi district, 2008

Characteristics	Individuals with TB symptoms **(*n *= 966)(%)**	PTB patients **(*n *= 40)(%)**	P-value	Total
**Population surveyed**				
Male	-	-		47 478
Female	-	-		24 430
Sex	-	-		23 038
Male	448 (46)	16 (40)	0.52	-
Female	518 (54)	24 (60)		-
**Age**				
15-24	139 (14)	4 (10)	0.62	-
25-34	230 (24)	9 (23)		-
35-44	197 (20)	8 (20)		-
45-54	201 (21)	6 (15)		-
55-64	129 (14)	9 (22)		-
> 65	70 (7)	4 (10)		-
**Occupation**				
Farmer	853 (88)	36 (90)		-
Other	72 (8)	2 (5)	0.82	-
Dependents	41 (4)	2 (5)		-
**Education**				
Illiterate	730 (75)	35 (88)	0.23	-
Elementary	199 (21)	4 (10)		-
Secondary +	37 (4)	1 (2)		-
**Marriage**				
Married	723 (75)	29 (72)	0.06	-
Single	131 (14)	3 (8)		-
Widowed	61 (6)	2 (5)		-
Divorced	51 (5)	6 (15)		-

### Data collection

The aim of the study was discussed with government authorities prior to the start of the data collection. After securing permission for the study, 34 Health Extension Workers (HEW) and 10 supervisors were recruited and trained to conduct data collection. The semi-structured questionnaire was pre-tested.

Before data collection commenced, the enumerators were assigned to one of 12 groups. Each group conducted a house-to-house survey among all households of the assigned study *kebele*. During the survey, the data collectors first asked the head of the household whether or not any family member had experienced a cough of more than 2 weeks duration, chest pain and shortness of breath. When such a case was found, the enumerators interviewed suspects using the questionnaire about TB symptoms, socio-demographic variables, the different health care actions taken and history of current or previous TB treatment. If the head of the household was not present during our initial visit the next responsible person in the family was interviewed. Repeat visits were conducted for those individuals who were not available during the first visit. After the interview every suspect was requested to provide a sputum sample for bacteriological analysis. Sputum samples were collected three times using the 'spot-morning-spot' method. The initial sputum was submitted on the spot. The second and third were collected the next day.

Supervision was provided throughout data collection. Ten field supervisors were assigned to monitor the daily activities. Specimens were placed in iceboxes and transported to the Regional Health Research Laboratory in Bahir Dar on the day of collection. Sputum microscopy was performed using the Ziehl-Neelsen hot staining procedure [14] by three experienced laboratory technicians. An independent reader was assigned to check the acid fast bacilli (AFB) results. Patients found to be AFB-positive during the survey were referred to the district TB control program for anti-TB chemotherapy.

### Definition of variables

*Health care action *was defined as any action taken by the individual patient to get relief from his/her symptoms. These included self-treatments, any visit to a traditional form of health care including the Orthodox Church for holy water treatment, local injectors or traditional healers, drug retail outlets (pharmacies, drug stores and rural drug vendors) or a visit to modern health care providers at public and private health facilities.

*Medical health providers *are modern health care facilities including health posts or clinics, health centres and hospitals owned by the government or the private sector.

*TB suspect *is an individual with a history of cough for 2 weeks or more, chest pain and difficulty of breathing. Subjects reported to have one or more of these symptoms by the head of the household were considered TB suspects.

### Statistical analysis

The analysis of health care seeking factors was done from two perspectives. We considered all kinds of health care actions and visits to medical providers only as two different outcome variables. The independent variables were socio-demographic and clinical factors.

Data were entered using the Statistical Package for the Social Sciences (SPSS) for Windows version 15.0. Proportions were calculated and logistics regression analysis was employed to assess associations between socio-demographic and clinical factors with the outcome variables. Initially a univariate analysis between the predictor and the outcome variables was performed. Thereafter variables that showed a p-value of less than 0.25 were further analysed by multivariable logistics regression analysis.

### Ethical approval

The Regional Committee for Medical Research Ethics in Eastern Norway (REK Øst) and the Ethiopian Science and Technology Agency in Addis Ababa, Ethiopia approved the study. All study participants provided their informed consent before the start of the study.

## Results

### Duration of cough

In this survey, a total of 1,006 (2.1%) individuals were identified as TB suspects of whom 40 were AFB-positives cases. The socio-demographic characteristics of the study population are presented in Table 1. Among the TB suspects, 542 (53.9%) women and 464 (46.1%) men had experienced a cough of more than two weeks duration, corresponding to a prevalence of 2.4% and 1.9%, respectively. These figures are calculated from a total eligible population of 23,038 women and 24,430 men. The median duration of cough was 43 weeks (inter-quartile range [IQR] 3.5-156 weeks). Four hundred sixty one (45.8%) of the suspects had a cough that lasted for 3 months to one year and 149 (14.9%) between 2 weeks to one month. Among the 40 patients identified with PTB during the survey, 7 (17.5%) had a cough of one month's duration or less and 22 (55%) between 3 months and one year (Table 2). One hundred twenty five (12.4%) of the TB suspects reported a past history of TB.

**Table 2:** Duration of cough among TB suspects and confirmed PTB cases in Merawi district, 2008

Duration	TB suspects *n *(%)	PTB cases *n *(%)	p-value
Two weeks	32 (3)	2 (5)	.58
Three weeks	35 (4)	2 (5)	
One month	75 (8)	3 (7)	
Three month	140 (14)	5 (12)	
Six month	102 (11)	8 (20)	
Nine month	50 (5)	3 (8)	
One year	147 (15)	6 (15)	
Above one year	385 (40)	11 (28)	

Total	966	40	

### Perception of illness and health care action

All study participants were asked about their perception regarding their illness. Surprisingly, 463 (46%) perceived their symptom as *"bird"*, a local expression referring to a disease believed to be caused by exposure to a wind or a cold weather. One hundred forty (14%) reported that their symptom was due to TB, 362 (36%) could not associate their symptom to any kind of illness, and the remaining 40 (4%) thought their symptoms to be due to asthma or the common cold.

**Figure 1:**
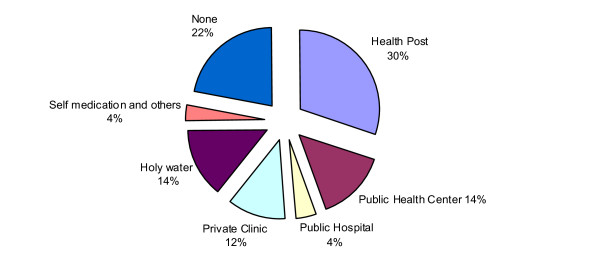
**Action taking among TB suspects (*n *= 1006)**.

**Figure 2:**
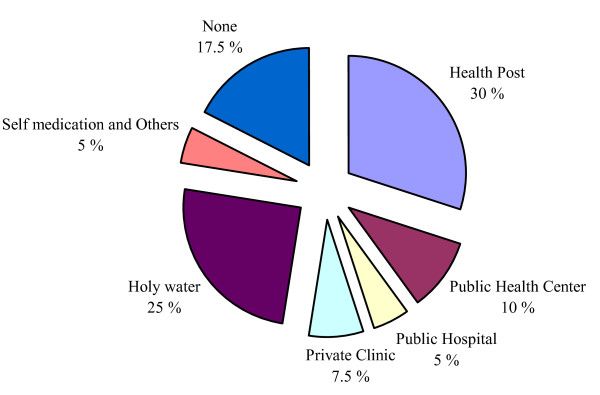
**Action taking among PTB patients (*n *= 40)**.

The majority, 787 (78%) of the TB suspects took different forms of health care actions for their symptoms. Six hundred four (60%) visited medical health providers including 307 (30%) who visited a health post or clinic that lacked diagnostic facilities for TB. One hundred eighty three (18%) sought traditional health care and 219 (22%) did not seek any form of health care for their symptoms (Figure 1). The median time of first health care action after the onset of symptoms was 30 days (IQR 12-68 days). Among the 40 PTB cases confirmed during the survey, 21 (52.5%) had received modern health care during the course of their illness, 12 (30%) opted for a traditional form of health care including self-treatment with holy water from the Orthodox Church and other sources, and 7 (17.5%) did not seek any form of health care. In general, health care seeking among PTB cases and TB suspects was almost similar except that many of the PTB cases opted for holy water (Figure 2).

Of the total 604 (60%) TB suspects who sought health care from medical health providers, only 140 (23%) reported having given a sputum sample for AFB during their visit. Among 307 (30%) TB suspects who visited a public clinic or health post, only 48 (15%) reported to have been referred to the next level of health care for further investigation and management. The majority of TB suspects were sent home with antibiotics or analgesics.

### Determinants of health care seeking

Taking all kinds of health care actions as an outcome variable, the results of logistic regression analysis showed that those with a cough lasting for more than 30 days (Adjusted Odds Ratio [AOR] 2, 95% Confidence Intervals [CI] 1.4-3.1), previous history of TB (AOR 1.8, 95% CI 1.1-3.0) and being dependents (AOR 0.39, 95% CI 0.2-0.7) were significantly associated with taking any kind of health care action for their symptoms. There was no difference in other socio-demographic variables (Table 3).

**Table 3:** Factors associated with health care seeking from any health provider among TB suspects in Merawi district, 2008

Factors	Total	Action taken *n *(%)	Crude Odds Ratio (95%CI)	Adjusted Odds Ratio	p-value
**Sex**					
Male	464	371 (80)	1	1	
Female	542	470 (75)	0.75 (0.6, 1)	0.78 (0.6, 1.0)	0.10
					
**Age**					
15-34	383	283 (74)	1	1	
35-54	412	330 (80)	1.42 (1.1, 2)	1.2 (0.9, 1.8)	0.81
55+	211	164 (78)	1.23 (0.8, 1.8)	1.0 (0.7, 1.6)	0.61
					
**Education**					
Illiterate	765	594 (78)	1	1	
Elementary	203	157 (77)	0.98 (0.7, 1.4)	0.9 (0.6, 1.5)	0.81
Secondary +	38	26 (68)	0.62 (0.3, 1.3)	0.8 (0.4, 1.8)	0.61
					
**Employment**					
Farmer	889	701 (79)	1	1	
Others	74	51 (69)	0.59 (0.4, 1)	0.68 (0.4, 1.2)	0.15
Dependents	43	25 (58)	0.37 (0.2, 0.7)	0.41 (0.2, 0.7)	0.007*
					
**Marriage**					
Married	752	590 (79)	1	1	
Single	133	101 (76)	0.87 (0.6, 1.3)	1.3 (0.8, 2.2)	0.33
Widowed	63	47 (75)	0.81 (0.5, 1.5)	1.2 (0.6, 2.2)	0.67
Divorced	57	38 (67)	0.55 (0.3, 0.9)	0.78 (0.4, 1.5)	0.45
					
**Cough Duration**					
≤ 30 days	149	95 (64)	1	1	
> 30 days	857	682 (80)	2.2 (1.5, 3.2)	2.0 (1.4, 3.1)	0.000*
					
**Distance**					
≤ 5 km	270	205 (76)	1	1	
> 5 km	736	572 (78)	1.1 (0.8, 1.6)	1.2 (.9, 1.7)	0. 40
					
**TB RX History**					
Yes	881	670 (76)	1	1	
No	125	107 (86)	1.87 (1.1, 3.2)	1.8 (1.1, 3.0)	0.032*
					
**Haemoptysis**					
No	352	277 (79)	1	1	
Yes	654	500 (76)	0.89 (0.64, 1)	0.89 (0.7, 1.2)	0.50
					
**Chest pain**					
Yes	887	691 (78)	1	1	
No	119	86 (72)	0.74 (0.48, 1.1)	0.7 (0.5, 1.2)	0.19
					
**Dyspnoea**					
Yes	818	634 (78)	1	1	
No	188	143 (76)	0.92 (0.63, 1.3)	0.95 (0.7, 1.4)	0.79

*Significant at < 0.05

When taking only medical health providers visit as an outcome variable, our analysis showed that women (AOR 0.8, 95% CI 0.6, 0.9) were found to be less likely to visit a medical health provider than men. Those with increased duration of cough (AOR 1.5, 95% CI 1.03, 2.1) and those with a previous history of TB (AOR 1.5, 95% CI (1.03, 2.3) were more likely to visit a medical facility than those with a shorter duration of cough and no history of TB. There was no difference in other socio-demographic variables (Table 4).

**Table 4:** Factors associated with health care seeking from medical provider among TB suspects in Merawi district, 2008

Factors	Total	Medical facility visit ***n*****(%)**	Crude Odds Ratio (95%CI)	Adjusted Odds Ratio	p-value
**Sex**					
Male	464	294 (63)	1	1	
Female	542	310 (57)	0.8 (0.6, 0.9)	0.8 (0.6, 0.9)	0.046*
					
**Age**					
15-34	383	220 (57)	1	1	
35-54	412	263 (64)	1.3 (0.9, 1.7)	1.2 (0.9, 1.6)	0.19
55+	211	121 (57)	0.9 (0.7, 1.4)	0.9 (0.6, 1.2)	0.56
					
**Education**					
Illiterate	765	455 (60)	1	1	
Elementary	203	127 (63)	1.1 (0.8, 1.6)	1.0 (0.7, 1.4)	0.77
Secondary +	38	22 (58)	0.9 (0.5, 1.8)	0.9 (0.5, 1.9)	0.97
					
**Employment**					
Farmer	889	544 (61)	1	1	
Other	74	38 (51)	0.7 (0.4, 1.1)	0.8 (0.5, 1.3)	0.30
Dependents	43	21 (51)	0.7 (0.4, 1.2)	0.8 (0.4, 1.4)	0.37
					
**Marriage**					
Married	752	458 (61)	1	1	
Single	133	79 (59)	0.9 (0.6, 1.4)	1.0 (0.7, 1.5)	0.92
Widowed	63	35 (56)	0.8 (0.4, 1.3)	0.9 (0.6, 1.6)	0.84
Divorced	57	31 (54)	0.7 (0.4, 1.3)	0.8 (0.5, 1.4)	0.55
					
**Cough Duration**					
≤ 30 days	149	77 (52)	1	1	
> 30 days	857	527 (62)	1.4 (1.5, 2.1)	1.5 (1.03 2.1)	0.035*
					
**Distance**					
≤ 5 km	270	155 (57)	1	1	
> 5 km	736	449 (61)	1.1 (0.8, 1.5)	1.3 (0.9, 1,8)	0.06
					
**TB RX History**					
No	881	518 (59)	1	1	
Yes	125	86 (69)	1.5 (1.03, 2.3)	1.5 (1.03, 2.3)	0.038*
					
**Haemoptysis**					
Yes	352	223 (63)	1	1	
No	654	381 (58)	0.8 (0.6, 1.0)	0.8 (0.6, 1.1)	0.25
					
**Chest pain**					
Yes	887	537 (61)	1	1	
No	119	67 (56)	0.8 (0.6, 1.2)	0.9 (0.6, 1.3)	0.49
					
**Dyspnoea**					
Yes	818	504 (62)	1	1	
No	188	100 (53)	0.7 (0.5, 0.9)	0.58 (0.9, 2.2)	0.15

* Significant at < 0.05

In this study 402 participants (40% of all TB suspects in the study) including 19 (47.5%) confirmed PTB cases did not consult medical health provider for their symptoms. The reasons for not consulting medical providers included the perception that the symptoms were not severe as well as other factors (Table 5).

**Table 5:** Reasons for not seeking health care from a medical facility among the study participants in Merawi district, 2008

Reasons	*n* (%)
Less severity of symptoms	166 (42)
Lack of money	130 (33)
Distance to health facility	55 (14)
Less belief in modern medicine	24 (6)
Lack of help/support	9 (2)
Pressure of work	12 (3)
Others	6 (2)

Total	402

## Discussion

In this study, 40% of the TB suspects and 47.5% of the PTB cases had not visited medical providers at the time of the survey. The major reason was that the study participants did not consider their symptoms to be severe. A similar finding from the Philippines reported that health care delay was attributed to an incorrect perception of symptoms and the high cost of medical care [15]. Another study also showed that personal health was not a priority in poor communities where people strive simply to meet their daily needs [16]. This may indicate that a number of factors including socio-economic and lack of information concerning TB play a key role in governing the health seeking behaviour of patients with TB symptoms.

On the other hand, among individuals who took health care actions, 60% visited medical health providers and of these only 30% had approached a medical provider that was equipped with a TB diagnostic facility. Peripheral medical health facilities in the study area lack diagnostic facilities for TB and are staffed by less experienced health workers (11). This implies that even though many TB suspects were able to visit a medical facility, the chance of getting a timely diagnosis was limited.

Our study showed that 78% of the TB suspects made one or more effort to seek health care from various sources. This indicates that patients seek help for their symptoms irrespective of the appropriateness of the source of health care. As reported in other studies, 51% of Korean [17] and 75% of Indian [3] symptomatic TB patients took health care actions for their symptoms. Our findings are similar to the results reported from India.

Our study showed a median duration of cough of 43 weeks implying a long infectious period. The finding is slightly higher than the 40 weeks median duration of cough observed in a study conducted in India [4,5].

In this study, 46% of the study subjects perceived their symptoms as "*bird"*. This may indicate a general lack of knowledge among the population about TB symptoms.

Among the socio-demographic factors, women were found to be less likely to visit medical providers for their symptoms than men. A study from Vietnam showed a long doctors' delay for female patients but did not indicate a difference in health seeking between men and women [18]. The reason for the delay in health seeking among women in our study may be related to the low status of Ethiopian women characterized by work overload and a "subservient role" to husbands and the family in general (19). Women are also left with few choices to make and do not have the right to exercise their own decisions [19]. Therefore, it is possible to view health care access for women as a reflection of this situation in Ethiopia. However, as gender is a broad issue, the reasons behind this difference should be the subject of further research.

Dependent individuals were less likely to take health care actions for their symptoms. As this group of study subjects included persons who depended on other individuals to meet their needs, they may have difficulty in accessing health care on their own due to financial constraints or physical incapabilities.

The association between previous history of TB treatment and visiting a medical provider suggests that the patient's motivation for seeking health care is influenced by an existing knowledge of the available services for TB. In contrast, patients with an increased duration of cough had a tendency to wait a long time before seeking medical attention. This results in a long infectious period that may increase the transmission of TB in the community.

Among the study subjects who consulted a medical provider only 23% reported having had a sputum microscopy test during their first visit. This implies that diagnostic tests for TB were not routinely requested or that the suspicion of TB and early referral by health providers was very low. This may be supported by the fact that many of the peripheral medical providers (health posts or clinics) in our study are staffed by new, inexperienced and over-burdened health workers [11]. Health workers employed at the mid-level health facilities also lack up-to-date training about TB case finding and case holding activities. In addition, attrition among the staff members is very high [11]. A study from a primary health care institution in India indicated that health workers assigned to case finding and case holding activities were often over-burdened and lacked ongoing support and training [20]. In another study from China, difficulties in understanding TB-related symptoms were observed among health workers, for example prolonged cough was perceived as a symptom of bronchitis rather than TB [21]. In our study, the probability of having had a sputum smear examination or being referred to the next level of health care was relatively high as 60% of the study participants had visited medical health providers.

Unlike the findings from India [4,5], the private health sector in our study was not consulted more often than public health facilities. This may be related to the very limited number of private facilities available in the study area, or that patients may have preferred the free of charge public health care over the private facilities.

In this study, we applied an active case finding strategy to detect TB suspects and patients in the community. We asked specific questions related to the presence of major TB symptoms such as cough. Household heads were asked to confirm the presence or absence of a TB suspect in their houses following a screening question posed by the interviewers. A study from Kenya in which a variety of active case finding strategies were applied had shown this method to be highly sensitive in identifying active TB cases in the community [22]. In another study from Kenya that reviewed hospital out-patients, the presence of weight loss and/or cough of between 1 and 12 months was found to be particularly sensitive [23]. In contrast, a recent study has shown that applying symptom inquiry such as cough alone for detecting suspected TB cases in a community may result in a high proportion of undetected TB cases [24]. This suggests the need for more research to further validate the available screening strategies.

Recently, varieties of survey strategies have been recommended to identify TB cases in community surveys [25]. These strategies are organized into four groups based on epidemiological importance and resource implications. These include 1) questionnaire interview followed by chest radiography, sputum smear examination and culture of all study participants, 2.) Screening by questionnaire followed by chest radiography and smear examination, 3) screening by questionnaire followed by chest radiography and 4) sputum smear examination of all study subjects [25]. The first three strategies are considered to be highly sensitive to detect TB cases. However, these strategies require sophisticated laboratory and/or chest radiography facilities. The last strategy is recommended for use in areas with limited infrastructures. In the present study, we employed symptom inquiry followed by sputum smear examination of all study participants taking into account the resource limitations in our study area.

The present study has limitations. First, our outcome measure of time of health care action taken was self-reported, implying a recall bias. To minimize this problem, we specifically asked about the onset of the major symptoms (cough) and how long did it take to consult a health provider after the onset of TB symptoms. In addition, we had used local calendar listing the main religious and national days to define the perceived date of onset of symptoms.

Secondly, symptom inquiry followed by sputum smear microscopy was solely used to identify TB cases. This screening strategy can not detect smear-negative and culture-positive TB cases (25), and as a result we may have missed many TB patients. However, we included several quality assurance methods to minimize the under detection of TB cases. Smear microscopy was performed by experienced laboratory technicians at the regional laboratory and an independent reader confirmed the results. Maximum care was taken during the collection and transport of sputum samples. Data collectors had received adequate training and supervision was provided throughout the survey. Thirdly, because of the relatively high prevalence of HIV in the study area, cases of HIV/TB coinfection may be misdiagnosed as a result of atypical clinical pictures. Therefore, our methodology is likely to underestimate the burden of active disease.

## Conclusion

This study showed that 78% of the participants had taken health care actions irrespective of the appropriateness of the source of health care seeking. Of these, 60% of the subjects had contacted a medical provider within a reasonable period of time following the onset of cough. However, most of the participants did not receive timely diagnosis and treatment for their symptoms. Therefore, accessing a simple and rapid diagnostic TB test at the health post level and involving all health providers in case finding activities are crucial interventions for early detection and treatment of TB cases. In addition, the provision of training for religious leaders of the Ethiopian Orthodox Church that addresses the medical consequences of TB and the importance of early health seeking among TB suspects may help in reaching a wider range of people in the community.

## Competing interests

The authors declare that they have no competing interests.

## Authors' contributions

SA and GB initiated the research, SY conducted the data collection, SY, GB, CHH and TY performed the data analysis and drafted the manuscript. The four authors edited and approved the final manuscript.

## Pre-publication history

The pre-publication history for this paper can be accessed here:

http://www.biomedcentral.com/1471-2458/9/454/prepub
